# Strip intercropping with local crops increased *Aconitum carmichaeli* yield and soil quality

**DOI:** 10.3389/fpls.2023.1147671

**Published:** 2023-03-03

**Authors:** Chen Liu, Pengdong Yan, Zhenyu Liu, Jianglan Zhang, Guoyan Zhang, Langjun Cui

**Affiliations:** ^1^ National Engineering Laboratory for Resource Development of Endangered Crude Drugs in Northwest China, The Key Laboratory of Medicinal Resources and Natural Pharmaceutical Chemistry, The Ministry of Education, College of Life Sciences, Shaanxi Normal University, Xi’an, China; ^2^ Chenggu County Qunli Traditional Chinese Medicine Cooperative, Chenggu, China

**Keywords:** *Aconitum carmichaeli* Debx., strip intercropping, agronomic traits, soil quality, TOPSIS, microbial communities

## Abstract

*Aconitum carmichaeli Debx*. is a traditional Chinese medicine that is cultivated in China and Japan. However, the monoculturing of this herb substantially decreases soil quality. Therefore, scientific planting management is crucial for resolving the current problems in the cultivation of *A. carmichaeli*. In this study, we conducted a comparative study on the soil environmental characteristics, herb growth and quality of *A. carmichaeli* intercropping with five local crops in two different areas. Herb growth and quality, including biomass and secondary metabolites, and rhizosphere soil environmental characteristics were measured. The results showed that the intercropping with the five local crops substantially improved the *A. carmichaeli* biomass and polysaccharide content, decreased the disease index, and altered three monoester diterpenoid alkaloids and three diester diterpenoid alkaloids accumulations. The intercrops also increased the soil pH, nitrogen-cycling-gene abundances, and potentially beneficial microorganism abundances, and it also changed the soil nutrient levels. Moreover, these intercropping patterns could alleviate the continuous cropping obstacles of *A. carmichaeli*. According to a comprehensive evaluation of the *A. carmichaeli* growth and quality, as well as the soil quality, the best intercropping systems were the *A. carmichaeli* intercropping with rice, maize, and peanut. In summary, the strip-intercropping systems could improve the *A. carmichaeli* growth and soil quality, and be beneficial to the sustainable ecological planting of *A. carmichaeli*.

## Introduction

Fuzi, which is the lateral root of *Aconitum carmichaeli* Debx., has been widely used in Asia as an essential herbal drug for 2,000 years (Fu et al., 2021). Researchers have dated the initial record on Fuzi back to the Han dynasty in the earliest Chinese classic medical work “Shennong Bencao Jing” (Singhuber et al., 2009; Zhou et al., 2015a). Fuzi is heat-processed into different traditional Chinese medicines (TCMs), and it is usually combined with other herbs in formulations to treat various diseases, such as rheumatism, cardiovascular diseases, painful joints, syncope, and bronchial asthma (Chen et al., 2020). Chuanwu, which is the main root of *A. carmichaeli*, is also one of the bulk Chinese medicinal materials. Alkaloids are the largest secondary metabolites in Fuzi and Chuanwu, and their types and contents directly affect the quality of these roots (Zhao et al., 2018). Monoester diterpenoid alkaloids have different pharmacological properties, such as neurotropic, antimicrobial, antitumor, hypotensive, analgesic, anti-inflammatory, muscle relaxant, antiarrhythmic, and local anesthetic properties (Sun et al., 2014). Diester diterpenoid alkaloids are toxic ingredients and are transformed into monoester diterpenoid alkaloids under high-temperature processing (Yang et al., 2014). The contents of monoester diterpenoid alkaloids and diester diterpenoid alkaloids are the standard compounds for the evaluation of the quality of the prepared pieces of Fuzi in *Pharmacopoeia of the People’s Republic of China* (2020 edition). Moreover, Fuzi polysaccharides, which are another pharmacological component, could stimulate murine lymphocyte proliferation and improve the immune function (Zhao et al., 2006). Due to their outstanding clinical effects and the considerable demand for Fuzi and Chuanwu in traditional Chinese medicine, more than eight provinces in China are currently cultivating *A. carmichaeli*, and the artificial planting regions have rapidly expanded in recent years (Fu et al., 2021).

*A. carmichaeli* prefers a sunny humid environment and sandy soil. In the main planting regions in China, farmers often plant *A. carmichaeli* in the first part of November and harvest it the following summer. From April to August, several diseases, including root rot and southern blight, frequently occur (Gao et al., 2021). In the past, farmers applied more chemical fertilizers and used high-density planting to obtain higher incomes, which strengthened the disease occurrence. Because *A. carmichaeli* planting could substantially decrease the soil pH and transform the soil microbial community structure, other local crop rotations have been proposed to improve the soil quality. However, with the increasing demand for this TCM and arable land restrictions in the main planting regions of China, the continuous cropping practice of *A. carmichaeli* has predominated, which has further decreased the herb yield, disturbed the rhizosphere soil microbial community, and altered the soil chemical parameters and pH (Xia et al., 2021). In recent years, more pesticides and chemical fertilizers have been applied to control the disease and improve the yields in artificially planted fields, which has decreased the herb quality and increased environmental pollution. Therefore, scientific planting management is crucial for resolving the current problems in the cultivation of *A. carmichaeli* in China (Yan et al., 2022).

Intercropping is the agricultural practice of cultivating two or more crops in the same field (Gao et al., 2019), and it is the practical application of basic ecological principles, such as diversity, competition, and facilitation (Schoeny et al., 2010). Provided that the intercrop components display differences in their competitive abilities for growth factors, such as light, water, and nutrients, the available environmental resources are more efficiently utilized, which results in yield improvements, quality maintenance, and disease control (Schoeny et al., 2010; Zhou et al., 2015b; Gao et al., 2019). Different intercropping systems, such as potato–legume (Gitari et al., 2020), corn–tobacco, wheat–tobacco (Zhou et al., 2015b), pea–cereal (Schoeny et al., 2010), and maize–soybean, have been widely used in sustainable farming. The ecophysiological mechanisms that underlie the intercropping performance adhere to three major principles, and different intercropping systems also have their own mechanisms. One of the reasons is likely the relaxation of the competition between species due to the spatial or temporal complementarity in the resource uptake. For instance, differences in the root and shoot architectural characteristics between species that grow together may lead to the complementary uptake of water or nutrients, light capture, and light-use efficiency, and especially when the component species are not simultaneously sown or harvested (Gitari et al., 2020; Bourke et al., 2021). Scholars have attributed the disease incidence reductions to host dilution, allelopathy, and microclimate and physical barrier effects (Zhou et al., 2015b; Bourke et al., 2021). In addition, intercropping could influence the soil microorganism communities and nutrient availabilities (Gao et al., 2019; Homulle et al., 2022). The performance and success of intercropping systems are affected by the crop choice, sowing proportions, and agronomic management. The crop choice is an important consideration that depends on the growing situation, local crop environment, suitability of the crop, and demand and availability of particular varieties (Maitra et al., 2021). For example, a wheat–fava bean intercrop substantially increased the productivity of two companion crops under subtropical conditions (Xu et al., 2019). In an *Avena sativa*–*Agropyron cristatum* intercropping system, the aboveground and total biomass of the intercropped *A. sativa* increased, while the biomass of the *A. cristatum* was not influenced (Liu et al., 2020a). The grain yield and economic value of the intercropped corn in a corn–tobacco intercropping system decreased when the relay-intercropping timing of the corn was delayed (Zhou et al., 2015b). Moreover, the different planting patterns of intercropping systems could increase or decrease the fruit quality or metabolic contents (Zhu et al., 2022). The intercropping of chestnut trees (*Castanea mollissima*) increased the tea length, weight, and quality by reducing the amino acid and catechin contents while increasing the anine and caffeine (Ma et al., 2017). In a *Brassica carinata*–*Solanum scabrum* intercrop, the total glucosinolate content in the *B. carinata* increased, while the majority of the kaempferol glycosides and hydroxycinnamic acid derivatives of both species decreased (Ngwene et al., 2017). The development of suitable cropping systems and achievement of potentially higher yields and better quality can be pursued under any agroclimatic conditions (Brooker et al., 2015).

In Jiangyou, which is one of the main planting regions in China, scholars explored the soil ecology and yield benefits of an *A. carmichaeli*–rice relay-intercropping pattern. According to the results, the intercropping system substantially increased the herb yield, soil pH, soil organic matter content, available nitrogen, available potassium, and root soil PLFA contents of bacteria, protozoa, and actinomycetes (Ren et al., 2018). The research provides an available intercropping pattern for *A. carmichaeli* planting. However, three issues still need to be resolved: (1) whether the *A. carmichaeli*–rice relay-intercropping pattern affects the herb quality; (2) the identification of the possible mechanism of the herb yield improvement; (3) whether there are other suitable intercropping patterns with the local crop. Thus, in this field study, we performed a comparative study on the soil environmental characteristics and Fuzi growth and quality of *A. carmichaeli* intercropping with five local crop varieties in two different areas. The objectives of this experiment were as follows: (1) to screen out suitable *A. carmichaeli* intercropping patterns, and (2) to uncover the possible ecophysiological mechanisms that underlie the intercropping performance. The outcomes of this study provide an appropriate *A. carmichaeli* intercropping system for sustainable medicinal planting models.

## Materials and methods

### Study site and experimental design

We conducted the experiment on October 20, 2020, in the main *A. carmichaeli* production regions: Jiangyou, Sichuan Province (31.77 N,104.67 E), and Chenggu, Shaanxi Province (33.15 N,107.33 E) (**Figures S1A, B**), which are located in a subtropical climate zone. Jiangyou has an annual temperature of 16°C, annual precipitation of 1055.5 mm, and an elevation of 532 m. Chenggu has an average annual temperature of 16.5°C, annual precipitation of 965 mm, and an elevation of 472 m.

The Qunli Traditional Chinese Medicinal Materials Cooperative of Chenggu (Chenggu, China) provided the seeds of maize (*Zea mays* L.), sesame (*Sesamum indicum* L.), peanut (*Arachis hypogaea*), mung bean (*Vigna radiata* (Linn.) Wilczek), and 45-day-old upland rice (*Oryza. sativa* L.). The fields in Jiangyou and Chenggu were previously cropped with rice and *A. carmichaeli*, respectively. We tilled the soil to a 30 cm depth, and we harrowed it without base fertilizer.

In Chenggu, we randomly selected plots with 30 cm heights and 1.25 m widths, and we separated them by trenches with at least 30 cm on each side. On each plot, we cultivated 3 rows of *A. carmichaeli* in a staggered manner, with plant and row spacings of 25 cm. In Jiangyou, farmers traditionally prune the roots to obtain larger Fuzi. Thus, in Jiangyou, the plots widths were 1.0 m, and we cultivated 2 rows of *A. carmichaeli* in a staggered manner. The other planting patterns were the same as those in Chenggu. We planted all the intercropping crops on the sides of the plots. We cultivated the control groups with *A. carmichaeli*. We present the sowing dates and intercrop planting patterns in **Table 1** and **Figures S1C, D**. During the growing period, we did not apply fertilizer or pesticide to the field.

**Table 1:** Sowing dates and intercrop planting patterns.

Treatment	Species	Intercropping Time	Plant Spacing	Location
J-maize	Maize	April 5, 2021	20 cm	Jiangyou
J-rice	Rice	June 1, 2021	20 cm	Jiangyou
J-control	/	/	/	Jiangyou
C-peanut	Peanut	May 1, 2021	20 cm	Chenggu
C-sesame	Sesame	April 5, 2021	20 cm	Chenggu
C-maize	Maize	April 5, 2021	20 cm	Chenggu
C-mung bean	Mung Bean	April 5, 2021	20 cm	Chenggu
C-control	/	/	/	Chenggu

### Sample collection

We collected the samples in Jiangyou and Chenggu on June 25 and July 25, 2021, respectively. Before the sampling, we surveyed the common diseases of *A. carmichaeli*, and we calculated the disease incidence (DI) according to Xia’s method (Xia et al., 2021).

We randomly selected more than 20 A*. carmichaeli* plants using the S-type sampling scheme in each treatment-group plot for the plant and rhizosphere soil sampling. After measuring the plant height (PH) and stem diameter (SD), we dug the plants out and discarded the attached soil. Then, we collected the rhizosphere soil with the help of a soft brush. We homogenized the soil samples in sterile plastic bags and then immediately transported them to the laboratory in an icebox. We stored one portion of the soil samples at -80°C for the DNA extraction, soil enzyme activity test, and abundance of bacteria, fungi, and nitrogen-cycling-gene determinations. We dried and sieved (< 2 mm) another portion of the soil sample at room temperature, and we then used it for the physicochemical analysis. We measured the aboveground biomass (AGB), Chuanwu weight (CW) (the weight of the main root of *A. carmichaeli*), and Fuzi weight (FW) (the weight of the lateral root of *A. carmichaeli*). The total underground biomass was the sum of the CW and FW. Some Fuzi were sampled and dried at 60°C to a constant weight, and were used for metabolites determination.

### Determination of Fuzi metabolites

We crushed the dry Fuzi into a powder and passed it through a 100-mesh sieve to determine the alkaloid and polysaccharide contents. We extracted approximately 1.0 g of the sample powder with 5 ml 0.05 M HCl for ultrasonic extraction over 30 min, and we then centrifuged it at 6000 rpm for 10 min. We collected 0.5 ml of the supernatant and diluted it with methanol to 2.5 ml, and we then further filtered it through a 0.22 μm syringe membrane filter. We used the filtrate for the determination of the alkaloid compounds. We analyzed the extracts on an Ultimate UHPLC PolarRP C18 (100 mm × 2.1 mm, 1.8 μm) column connected to a Waters UPLC H-Class ultra-high-performance liquid chromatography system. The mobile phase comprised A (acetonitrile) and B (2 mM ammonium acetate containing 0.1% formic acid), and we filtered the solvent through a 0.22 μm filter. We used a gradient elution at a flow rate of 0.4 ml/min. The gradient elution program of the A mobile phase was as follows: 0.01–5min, 20%; 6 min, 25%; 8 min, 30%; 9 min, 20%; 11 min, 20%. We maintained the column temperature at 35°C. We set the detection wavelength at 240 nm. The sample injection volume was 10 μl. We referenced Chen’s method (Chen et al., 2020) for the major peak identification, standard solution preparation, and methodology validation.

We purchased six diterpenoid alkaloids (benzoylhypaconitine, benzoylaconitine, benzoylmesaconitine, hypaconitine, aconitine, and mesaconitine) from the Chengdu Must Bio-technology Co. Ltd (Chengdu, China). The monoester diterpenoid alkaloid content was the sum of the benzoylhypaconitine, benzoylaconitine, and benzoylmesaconitine alkaloids. The diester diterpenoid alkaloid content was the sum of the hypaconitine, aconitine, and mesaconitine alkaloids. The total diterpenoid alkaloid content was the sum of the total monoester and total diester diterpenoid alkaloids. We determined the polysaccharide content using the phenol sulfuric acid method (Xia et al., 2015).

### Determination of soil physicochemical parameters

We determined the soil pH value, NH_4_^+^-N, NO_3_^-^-N, phosphate, and potassium oxide contents, and medium and trace elements in the soil according to Xia’s method (Xia et al., 2021). We determined the soil organic matter content using the potassium dichromate volumetric method (Wu et al., 2014).

We used 0.5 g of *A. carmichaeli* rhizosphere soil samples as the initial material for the total DNA extraction using the PowerSoil DNA Isolation Kit (MoBio, USA), according to the manufacturer’s protocols. We detected the genomic DNA quality by agarose gel electrophoresis, and we determined the concentration using a NanoDrop (Thermo Scientific, Wilmington, USA) (Zeng et al., 2020). We quantified the bacterial 16S rRNA gene, fungi ITS, nitrification- and denitrification-process enzyme genes (*amoA*, *nxrA*, *narG*, *nirS*, *norB*, *nosZ*), and nitrogenase iron protein subunit gene (*nifH*) using a real-time PCR detection system (StepOnePlus, ABI) (Zhao et al., 2017). For the qPCR, we added 10 ng of the template soil DNA, 0.5 μl of primers (10 mM), 0.5 μl of ROX Reference Dye II (50×), 10 μl of 2 × SYBR^®^ Premix Ex TaqTM (Takara), and sterile deionized water to a total 20 μl reaction volume. We present the primer sequences and PCR protocol in **Table S1**. The Sangon Biotech (Shanghai) Co., Ltd. (Shanghai, China) synthesized the primers and target gene sequences. We added the target gene sequences to the *BamH*1 and *EcoR*1 restriction sites, and we then cloned them to the UC-57 vector and transformed them into *Escherichia coli* Top10. We restricted the isolated cloned plasmids with *EcoR*1, and we created a standard curve by the tenfold serial dilution of the target gene (10 ng/μl-10^-5^ ng/μl) (Wu et al., 2012). We calculated the gene abundances as previously described.

We determined the soil catalase activity by potassium permanganate titration, the soil invertase activity by 3,5-dinitrosalicylic acid colorimetry, the urease activity by sodium phenolate sodium hypochlorite colorimetry, and the acid phosphatase activity by the colorimetric method of disodium phenylphosphate (Zhao et al., 2022).

### TOPSIS comprehensive evaluation

We used the technique for order preference by similarity to ideal solutions (TOPSIS) to identify the best alternative for achieving higher crop yields and better crop and soil qualities for *A. carmichaeli* intercropping systems (Rathore et al., 2021).

For the TOPSIS comprehensive evaluation of the herb yield and quality, we screened six key original evaluation parameters, and we assigned them different weighing values according to Yue’s research results (Yue et al., 2014), the medicinal parts, and the pharmacological activities. We present the screened key original evaluation parameters and the values of their attributes in **Table S2**. After determining the positive and negative ideal solutions and calculating the Euclidean distance, we calculated the comprehensive benefit evaluation index (*C_i_*) for all the treatments.

In this study, the contents of most of the soil medium and trace elements were not remarkably varied among the different treatment groups. The abundances of the total bacteria and fungi were the indicator of the whole community; however, their size changes did not accurately correspond to the soil quality. Among the soil parameters, the pH value, levels of organic matter, nitrogen, and potassium, nitrogen-cycling-gene abundance, and soil enzyme activity were substantially correlated to the plant growth. Thus, we included these indicators in the evaluation system. Among these soil characteristics, only the pH value is the interval index, and it needs to be positively ideally transformed. According to the GB/T 23399-2009 standard, the optimum growth soil pH value for *A. carmichaeli* is 7–8. In this study, all the soil pH values were lower than 7. We transformed the pH values using the following equation:


M=7−min(x)

$$\hat{x_{i}}=1-\frac{7-x_{i}}{M}=\frac{x_{i}-\text{min}\left(x\right)}{7-\text{min}\left(x\right)}$$

where *x* is the soil pH value; *M* is the medium parameter; *x_i_* is the pH value before the transformation; $\hat{x_{i}}$ is the pH value after the transformation. Complete algorithm come from https://zhuanlan.zhihu.com/p/266689519.

In this study, we analyzed a total of 28 soil parameters. Among these, the pH, organic matter, nitrogen, phosphorus, and potassium are vital for plant growth and are strongly affected by intercropping patterns. The abundances of nitrogen-cycling genes and enzymes could regulate the soil nutrient cycling and organic matter degradation. Although both medium and trace elements are also vital for plant growth, most of their contents in the different intercrops were not substantially influenced. The 16S rDNA and ITS abundances reflected the abundances of the soil total bacteria and fungi. However, they did not reflect the soil quality. Thus, we screened a total of 17 parameters to comprehensively evaluate the soil quality: pH; organic matter; NH_4_^+^-N; NO_3_^-^-N; phosphate; K; *amoA*; *nxr*; *narG*; *nirS*; *norB*; *nosZ*; *nifH*; catalase; invertase; urease; acid phosphatase. We present their attribute values in **Table S3** (Yue et al., 2014). After determining the positive and negative ideal solutions and calculating the Euclidean distance, we calculated the comprehensive benefit evaluation index (*C_i_*) for all the treatments.

### Metagenomic analysis of soil microorganisms

We used the *A. carmichaeli* rhizosphere soil samples of the J-maize, J-rice, and J-control to analyze the microbial communities. For each sample, we extracted 500 mg of soil sample with the MP FastDNA TM SPIN kit for Soil, and we fragmented 0.2 ug of quality-checked DNA into 450 bp by Covaris M220 for the library preparation. We subsequently performed the library preparation using the TruSeq DNA PCR-Free Library Preparation Kit, and we sequenced it on the Illumina NovaSeq 6000 platform (Illumina Inc., San Diego, CA, USA) in BIOZERON Biotechnologies Inc., Shanghai, China. We quality-controlled each raw fastq file with a size of 10 Gb by fastp with default parameters by removing the sequencing adapters and low-quality reads (Chen et al., 2018). In total, we acquired from 99.90% to 100% clean reads per sample (**Table S4**). First, we used kraken2-build to download seven databases: viral, UniVecCore, plasmid, protozoa, fungi, archaea, bacteria. Then, we built the kraken2-annotation library, and we used the bracken-built library to build the bracken-annotation library. Subsequently, we used kraken2 with a parameter confidence equal to 0.1 for the preliminary annotation (Wood et al., 2019), and we used bracken with the parameters THRESHOLD = 10 and READ_LEN = 150 for the secondary species annotation (Lu et al., 2017). We performed all the above data analyses in parallel using parallel software (Tange, 2011). After obtaining the species abundance table, we used the vegan package to analyze the β-diversity, the psych package to obtain the sample similarity to confirm the repeatability of each subsample, the vegan package to obtain the species α-diversity, the ggplot2 package to display the species distribution, and LEfSe (version 1.1.2) software (Segata et al., 2011), the VennDiagram R package, and some base functions to analyze the differential species. We performed all these analyses in R (version 4.0.5, https://cran.r-project.org/ ) (see **Table S5** for the R package used in this process).

### Data analysis

The data was analyzed in the R (version 4.0.5, https://cran.r-project.org/) (Robinson et al., 2010; McMurdie and Holmes, 2013; Love et al., 2014; Paradis and Schliep, 2019; Villanueva and Chen, 2019) and OmicStudio(Lyu et al., 2023). All the tests were performed at least three times. Correlation analyses were performed using advanced chord chart (pearson, p<0.05) (Metware Cloud). Partial least squares path model (PLS-PM) was conducted in the R (Liao et al., 2019). The results were expressed as means ± SE values, and the data was analyzed using ANOVA analysis.

## Results

### Agronomic performance and active ingredient accumulations

**Figure 1:**
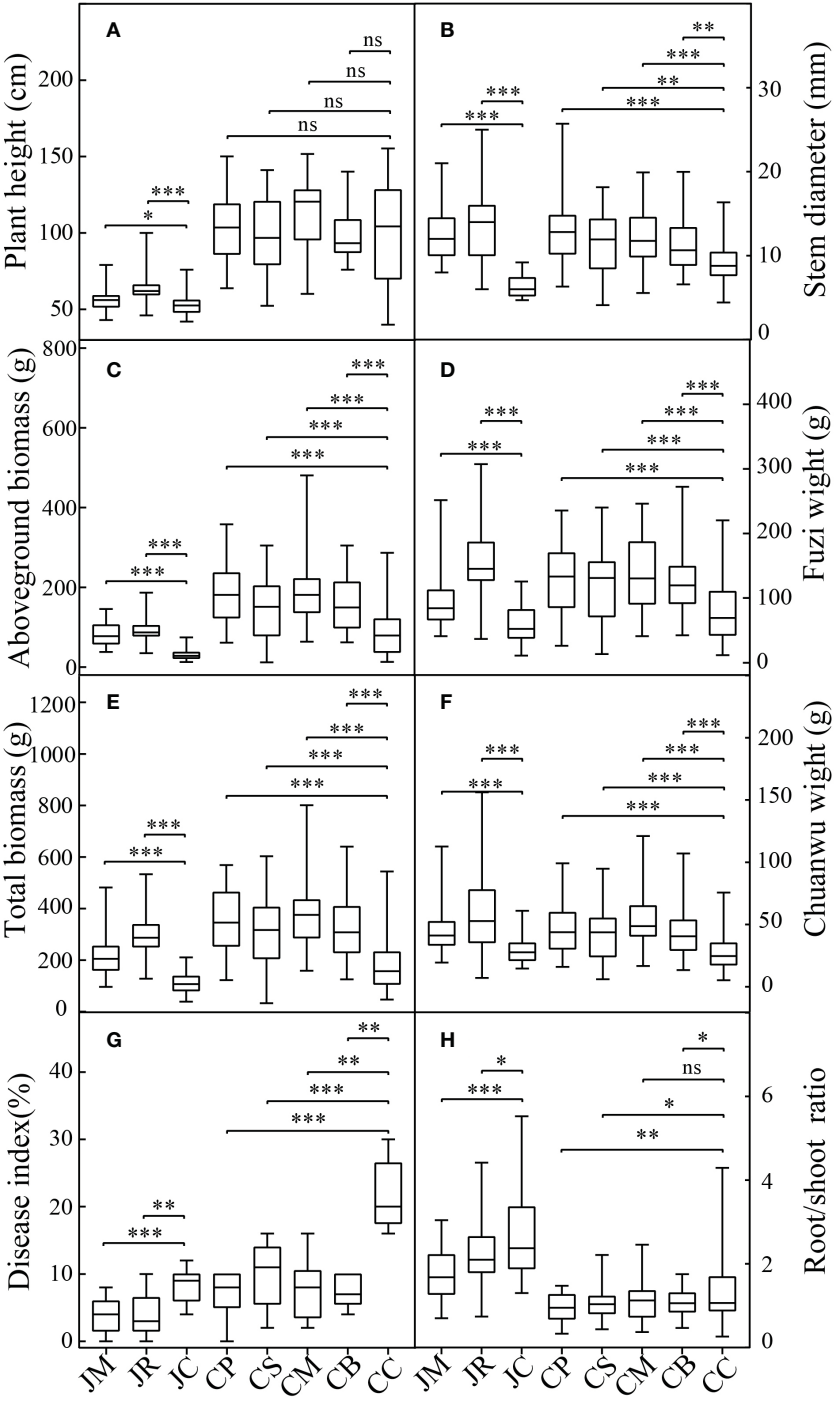
Agronomic performance of different strip-intercropping modes: **(A)** plant height; **(B)** stem diameter; **(C)** aboveground biomass; **(D)** Fuzi weight; **(E)** total weight; **(F)** Chuanwu weight; **(G)** disease index (%); **(H)** root/shoot ratio. Data are the means ± SD from 45 biological replicates. Asterisks (*) indicate significant differences (*p<0.05, **p<0.01, ***p<0.001 based on t-test.), "ns" indicate no significant difference. (JM: *A. carmichaeli*-maize strip-intercropping in Jiangyou region; JR: *A. carmichaeli-*rice strip-intercropping in Jiangyou region; JC: *A. carmichaeli* monocropping in Jiangyou region; CP: *A. carmichaeli-*peanut strip-intercropping in Chenggu region; CS: *A. carmichaeli-*sesame strip-intercropping in Chenggu region; CM: *A. carmichaeli*-maize strip-intercropping in Chenggu region; CB: *A. carmichaeli-*mung bean strip-intercropping in Chenggu region; CC: *A. carmichaeli* monocropping in Chenggu region. The same below).

The agronomic characteristics and effective component contents of *A. carmichaeli* were present in **Figure 1**. Except for the plant heights of the C-mung bean, C-sesame, and C-peanut, the plant height, stem diameter, aboveground biomass, Fuzi biomass, Chuanwu biomass, and total biomass of the intercropping of *A. carmichaeli* with the five crops in both regions were substantially increased. The increases in the aboveground biomass, underground biomass, Fuzi biomass, and Chuanwu biomass ranged from 83.3%–200.8%, 59.2%–149.6%, 58.3%–178.5%, and 44.8%–103.6%, respectively. Especially for the medicinal part of Fuzi, the highest increment treatment was the J-rice, followed by the C-maize and C-peanut. For the medicinal part of Chuanwu, the three highest increment treatments were the C-maize, J-rice, and C-peanut. Moreover, except for the Chuanwu biomass, the average increments of the plant height, stem diameter, aboveground biomass, underground biomass, and Fuzi biomass in Jiangyou were higher than those in Chenggu. Moreover, the intercropping decreased the *A. carmichaeli* disease indexes and root/shoot ratios, with many of them reaching significant levels.

**Figure 2:**
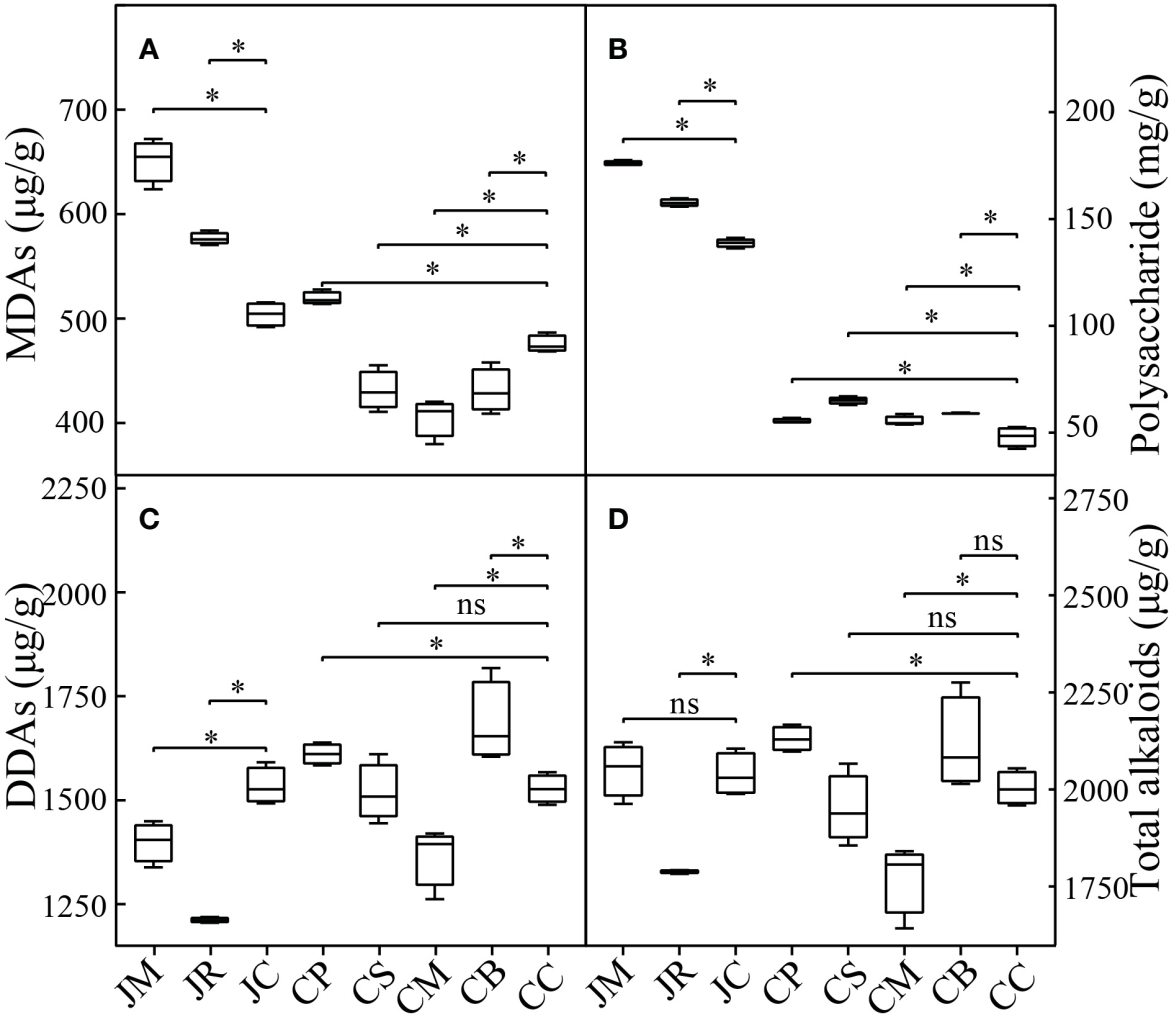
Active ingredient accumulations of different strip-intercropping modes: **(A)** monoester diterpenoid alkaloids (MDAs) content; **(B)** polysaccharide content; **(C)** diester diterpenoid alkaloids (DDAs) content; **(D)** total diterpenoid alkaloids content. Data are the means ± SD from 4 biological replicates. Asterisks (*) indicate significant differences (*p<0.05 based on t-test), "ns" indicate no significant difference.

The main secondary metabolite accumulations of the Fuzi were differently affected by the intercropping practices (**Figure 2**). In the J-maize and J-rice, the contents of Fuzi polysaccharides and total monoester diterpenoid alkaloids were remarkably improved, and the total diester diterpenoid alkaloids substantially decreased. The total diterpenoid alkaloids of the J-rice decreased, while the J-maize was not substantially affected. These metabolite accumulations of the *A. carmichaeli* intercropping in the Chenggu region had a different trend from those in Jiangyou. The contents of the total monoester diterpenoid alkaloids in the C-peanut increased, and they decreased in the C-sesame, C-maize, and C-mung bean (*p*< 0.05). For the total diester diterpenoid alkaloids and total diterpenoid alkaloids accumulations, the intercropping with peanut and mung bean increased them, the intercropping with maize decreased them (*p*< 0.05), while the intercropping with sesame did not substantially vary them. Moreover, the intercropping with four crops in Chenggu increased the Fuzi polysaccharide accumulation.

Specifically, the three monoester diterpenoid alkaloids accumulations of the intercropping *A. carmichaeli* in the two regions had similar variants. The benzoylhypaconitine contents were substantially increased, while the benzoylaconitine and benzoylmesaconitine contents were substantially decreased. However, the three diester diterpenoid alkaloids accumulations of the *A. carmichaeli* intercropping in the two regions had different variants. In Jiangyou, the hypaconitine contents in all the intercropped *A. carmichaeli* were remarkably increased, while the aconitine and mesaconitine contents were decreased. However, in Chenggu, the hypaconitine contents in the four *A. carmichaeli* intercrops were decreased. The aconitine and mesaconitine accumulations in the C-maize decreased, while they increased in the other three intercrops (**Table S6**).

### TOPSIS comprehensive evaluation for better herb yield and quality

According to the evaluation of the *A. carmichaeli* growth and quality under these treatments, the comprehensive benefit evaluation index (*C_i_*) varied from 0.181 to 0.821 (**Table 2**). The best intercropping system was the J-rice, followed by the J-maize, C-peanut, and C-maize, with *C_i_* values of 0.821, 0.625, 0.500, and 0.496, respectively. The *C_i_* values of both the J-rice and J-maize were all higher than those of the other intercropping practices in the Chenggu region. In addition, the evaluation with the “Fuzi weight” and “Monoester diterpenoid alkaloids” as the main indicators were conducted, and results were similar to the previous text (**Table S7**).

**Table 2:** TOPSIS comprehensive evaluation score ranking of *A. carmichaeli* growth and quality parameters.

Treatments	*D_i_* ^+^ *	*D_i_* ^-^ *	*C_i_* *	Rankings
J-maize	0.051	0.085	0.625	2
J-rice	0.023	0.106	0.821	1
J-control	0.090	0.050	0.357	7
C-peanut	0.068	0.068	0.500	3
C-sesame	0.076	0.055	0.419	6
C-maize	0.076	0.075	0.496	4
C-mung bean	0.075	0.062	0.452	5
C-control	0.102	0.022	0.181	8

*D_i_ ^+^, *D_i_* ^-^: distance between each alternative and positive and negative ideal solution, respectively. *C_i_*: comprehensive benefit evaluation index.

### Soil physical and chemical properties and functions

**Figure 3:**
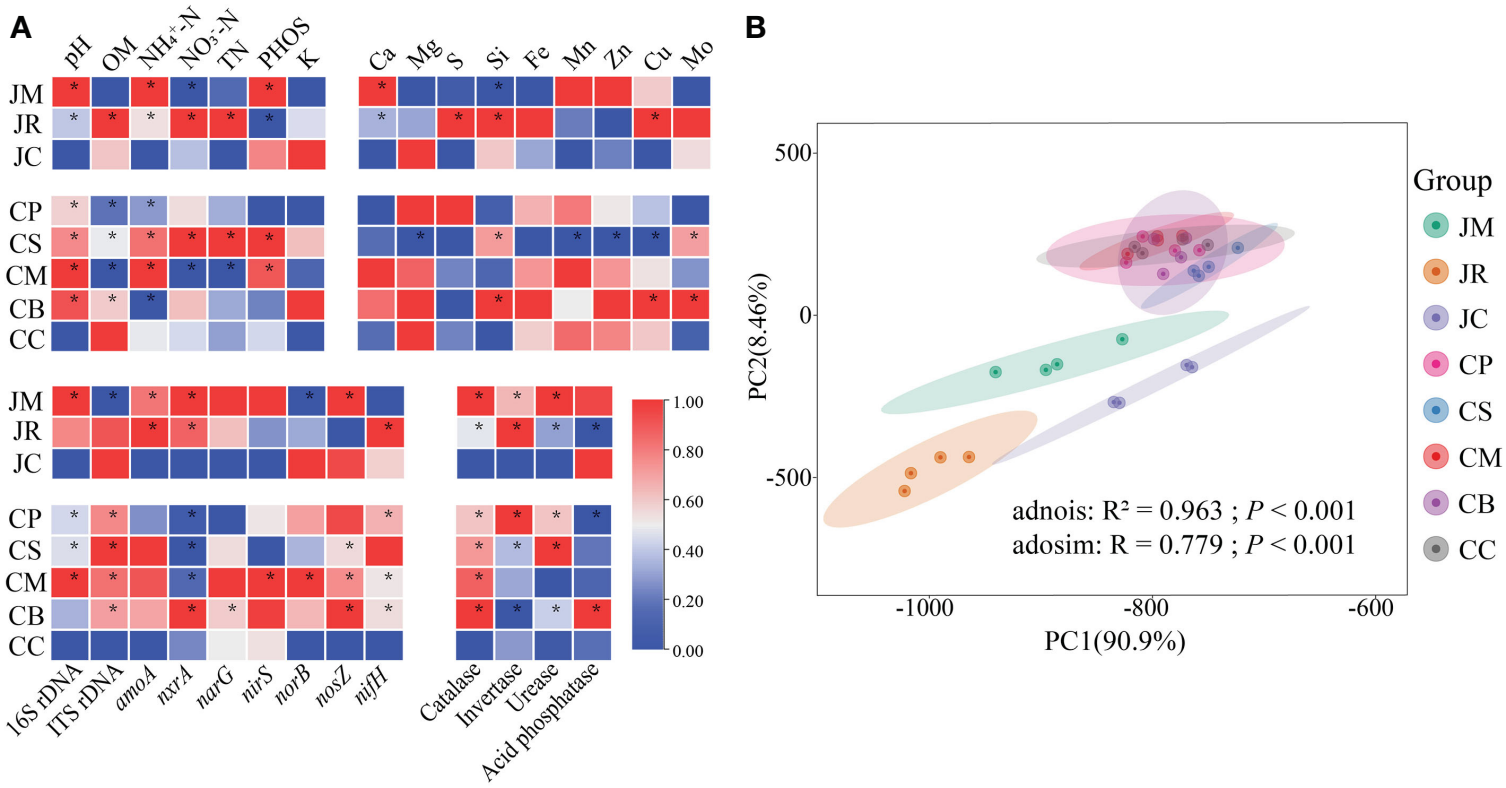
Overview of soil physicochemical properties and soil functions of different strip-intercropping modes: **(A)** heatmap of soil physicochemical properties and soil functions, four replicates were used for each sample, asterisks (*) indicate significant differences (*p<0.05 based on t-test); **(B)** principal component analysis (PCAs) of all soil properties (Adonis and Anosim test). (OM, organic matter; TN, total nitrogen; PHOS, phosphate).

In the two regions, the intercropping remarkably increased the soil pH, and the average increments were 8.8% and 14.9% in Jiangyou and Chenggu, respectively. Compared to the monoculture group, the soil organic matter content of the C-peanut, C-sesame, C-maize, and C-mung bean were substantially decreased, while the J-rice remarkably increased, and the J-maize did not vary. For the soil NH_4_^+^-N, the J-rice, J-maize, C-sesame, and C-maize were remarkably increased, while the C-mung bean and C-peanut were remarkably decreased. For the soil NO_3_^-^-N, the J-maize and C-maize were remarkably decreased, the J-rice and C-sesame increased, while the other groups did not vary. For the soil phosphate, the J-maize, C-maize, and C-sesame were remarkably increased, the J-rice decreased, and the C-mung bean and C-peanut did not vary. Furthermore, none of the intercropping systems had any substantial effects on the K^+^, while the C/N ratios were all decreased (**Figure 3A**).

Intercropping with the five local crops could not strongly affect the contents of the most of soil medium and trace elements, which only the Si content of the J-maize, the Mg^2+^, Mn^2+^, Zn^2+^, and Cu^2+^ levels of the C-sesame were remarkably reduced. Moreover, intercrops improved most of the abundance of the 16S rDNA, ITS rDNA, and nitrogen cycling related genes. For example, the ITS rDNA and *norB* abundances of the J-maize, the *nxrA* abundance in the C-peanut, C-sesame, and C-maize were remarkably decreased, while the 16S rDNA, ITS rDNA, *amoA*, *nxrA*, *nirS*, *norB*, *narG*, *nosZ*, and *nifH* abundances in these intercropping patterns were either remarkably increased or not affected (**Figure 3A**).

For the soil enzymes, only the invertase activity in the C-mung bean and the acid phosphatase in the J-rice and C-peanut was decreased, and the catalase, urease, invertase, and acid phosphatase in these intercropping systems were either strongly increased or not affected (**Figure 3A**). According to the PCA analyses of all these soil properties, the difference between the groups was substantially greater than that within the groups, and the two regions were considerably separated (**Figure 3B**).

### TOPSIS comprehensive evaluation for better soil quality

According to the evaluation of the *A. carmichaeli* soil quality under the eight treatments using TOPSIS, the comprehensive benefit evaluation index (*C_i_*) varied from 0.212 to 0.751 (**Table 3**). The best intercropping system was the J-maize, followed by the J-rice, C-sesame, and C-peanut, with *C_i_* values of 0.751, 0.554, 0.497, and 0.464, respectively. The *C_i_* values of both the J-rice and J-maize were higher than those of the other intercropping practices in the Chenggu region.

**Table 3:** TOPSIS comprehensive evaluation score rankings of different soil samples.

Treatment	*D_i_* ^+^ *	*D_i_* ^-^ *	*C_i_* *	Ranking
J-maize	0.297	0.896	0.751	1
J-rice	0.581	0.721	0.554	2
J-control	0.889	0.403	0.312	7
C-peanut	0.618	0.535	0.464	4
C-sesame	0.603	0.597	0.497	3
C-maize	0.747	0.566	0.431	6
C-mung bean	0.719	0.611	0.460	5
C-control	0.889	0.239	0.212	8

*D_i_ ^+^, *D_i_* ^-^: distance between each alternative and positive and negative ideal solution, respectively. *C_i_*: comprehensive benefit evaluation index.

### Correlation between agronomic performance and soil properties

**Figure 4:**
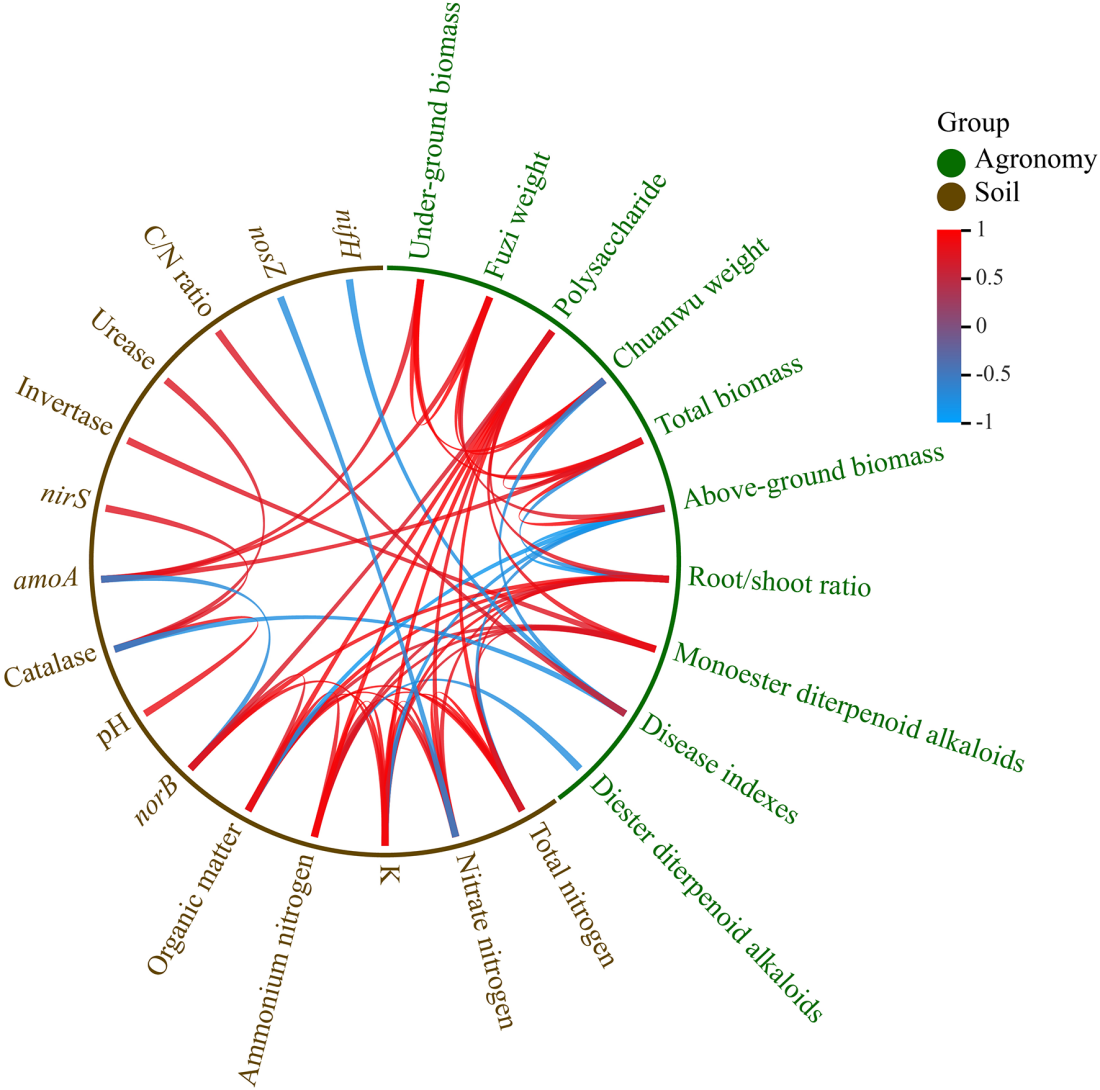
Correlation between agronomic performances and soil properties (pearson, *p<*0.05).

According to the correlation analysis between the soil and plant variables, the aboveground biomass was positively corelated with the pH and *amoA* but was negatively correlated with the organic matter, NH_4_^+^-N, NO_3_^-^-N, total nitrogen, K^+^, and *norB*. The biomasses of the underground, Fuzi, and Chuanwu were positively correlated with the pH and *amoA*. The total monoester diterpenoid alkaloids were positively related to the soil organic matter, NH_4_^+^-N, NO_3_^-^-N, total nitrogen, K^+^, and invertase. The total diester diterpenoid alkaloids were negative correlated to the NH_4_^+^-N, and they were positively correlated with the invertase and acid phosphatase. The disease index was remarkably positively related to the C/N ratio, while it was negatively related to the catalase, *nifH*, and pH. Most of these correlation ships reached significant levels (**Figure 4**).

### Metagenomic analysis of soil microorganisms

**Figure 5:**
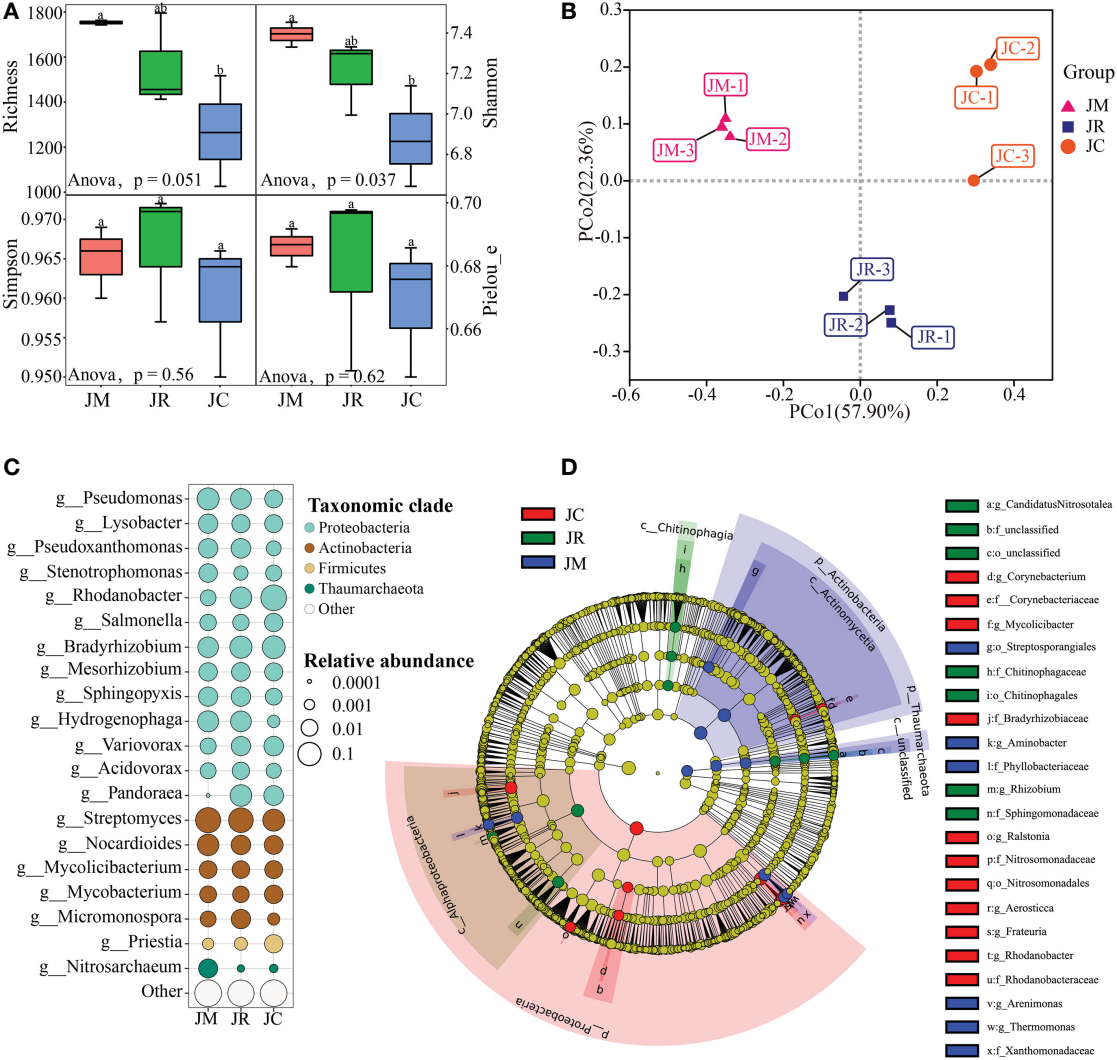
**(A)** α (Anova analysis, wilcox. test: *p<*0.05) and **(B)** β diversities of soil microbial communities at sites after strip intercropping in Jiangyou, and **(C)** relatively rich dominant genera in the top 20 and **(D)** LEfSe analyses with different abundances between soil samples.

We obtained a total of 2,767 high-quality sequences from all the monoculture- and intercropping-system rhizosphere soil samples. We grouped the sequences into 2,665 bacterial OTUs, 38 fungal OTUs, and 34 archaea OTUs (**Table S8**). For the soil microbial communities, the richness, Shannon, Simpson, and Pielou of the J-rice and J-maize were increased, and the increments of the richness and Shannon reached significant levels (**Figure 5A**). At the phylum, genus, and species levels, the J-rice, J-maize, and J-control had 22, 434, and 1,185, respectively (**Figure S2**). In the three rhizosphere soil samples, the bacteria were dominant, the abundance of which accounted for more than 96%. Fungi and archaea mainly appeared in the J-control and J-maize, respectively (**Figure S3**). Proteobacteria were the dominant phyla, which accounted for at least 50% of the three soil samples (mainly in the J-control). The subsequent dominant phyla were Actinobacteria (mainly in the J-rice and J-maize) and Firmicutes (mainly in the J-control). The three dominant genera were *Streptomyces* (mainly in the J-rice and J-maize), *Rhodanobacter* (mainly in the J-control), and *Bradyrhizobium* (mainly in the J-rice and J-control) (**Figures S4**, **S5**).

According to the principal coordinate analysis (PCoA), the rhizosphere soil of the control, J-maize, and J-rice had substantially different microbial beta diversities (**Figure 5B**). According to the LEfSe analysis results, there were 1,278 different markers at all the classification levels (*p*< 0.05 by Wilcox test), including 577 with LDA values greater than 2, 159 with LDA values greater than 3, and 24 with LDA values greater than 4 (**Table 4**).

**Table 4:** Overview of number of differences in LEfSe.

Number of Different Markers	ALL	Kingdom	Phylum	Class	Order	Family	Genus	Species
Total Difference	1278	1	6	25	42	93	264	847
LDA > 2	577	1	5	14	24	52	132	349
LDA > 3	159	1	4	9	15	25	40	65
LDA > 4	24	1	3	3	2	5	3	7

Based on the LEfSe results, the Streptosporangiales order, the Xanthomonadaceae and Phyllobacteriaceae families, and genera including *Thermomonas*, *Arenimonas*, and *Aminobacter* contributed to the microbial community composition characteristics of the J-maize soil sample. The Chitinophagales order, Sphingomonadaceae and Chitinophagaceae families, and genera of *Rhizobium*, *Candidatus*, and *Nitrosotalea* contributed to the microbial community composition characteristics in the J-rice soil sample. Finally, the Nitrosomonadales order, Rhodanobacteraceae, Nitrosomonadaceae, Bradyrhizobiaceae, and Corynebacteriaceae families, and genera including *Rhodanobacter*, *Frateuria*, *Aerosticca*, *Ralstonia*, and *Corynebacterium* contributed to the microbial community composition properties of the J-control soil sample (**Figures 5C, D**).

According to the comprehensive survey results, 178 dominant microorganisms of 28 genera in the two intercropping systems were potentially beneficial ones. The 28 genera were as follows: *Agromyces*; *Aminobacter*; *Azorhizobium*; *Rhizobium*; *Jeotgalicoccus*; *Diaphorobacter*; *Collimonas*; *Amycolatopsis*; *Streptomyces*; *Kosakonia*; *Zobellella*; *Haematobacter*; *Pontibacter*; *Ancylobacter*; *Frigoribacterium*; *Rhodococcus*; *Nitrososphaera*; *Burkholderia*; *Cellulomonas*; *Flavobacterium*; *Glutamicibacter*; *Hartmannibacter*; *Lysobacter*; *Massilia*; *Microbacterium*; *Micromonospora*; *Pseudomonas*; *Raoultella.* Moreover, among the 68 dominant microorganisms in the J-control, we identified 15 potentially beneficial genera and 6 harmful genera. The beneficial genera were as follows: *Rhodoplanes*; *Sporolactobacillus*; *Sulfuricurvum*; *Brevundimonas*; *Desulfitobacterium*; *Desulfosporosinus*; *Rahnella*; *Priestia*; *Nitrosomonas*; *Acetobacter*; *Cedecea*; *Cohnella*; *Dyella*; *Frateuria*; *Rhodoferax.* The pathogenic genera were as follows: *Winogradskyella*; *Xanthomonas*; *Ewingella*; *Rathayibacter*; *Proteus*; *Ralstonia* (**Figures 5C, D**).

### Correlation between microbial communities and soil properties

**Figure 6:**
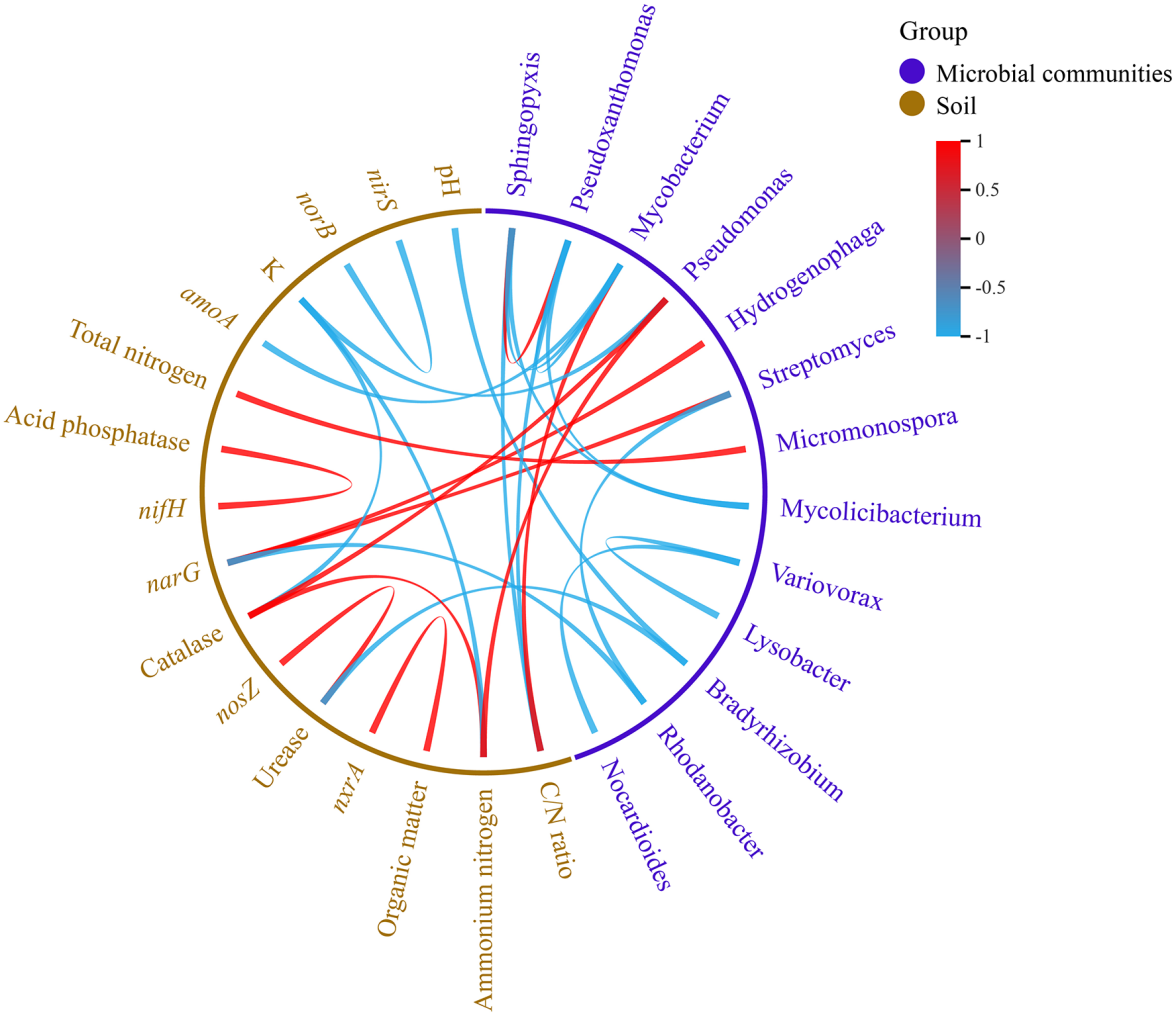
Correlation between microbial communities and soil properties (pearson, *p<*0.05).

According to the correlation analysis between the microbial communities and soil variables, *Bradyrhizobium* was negatively correlated with the pH and urease. *Streptomyces* and *Hydrogenophaga* were positively correlated with the *narG*, but *Rhodanobacter* was negatively correlated with the *narG*. *Pseudomonas* was negatively correlated with the NH_4_^+^-N and catalase, but was negatively correlated with the K^+^. *Mycobacterium* was negatively correlated with the C/N ratio, but was negatively correlated with the *amoA*. *Pseudoxanthomonas* and *Sphingopyxis* were positively correlated with the C/N ratio. *Micromonospora* was positively correlated with the total nitrogen. These correlation ships reached significant levels (**Figure 6**).

### Relationship between soil, nitrogen cycle gene, enzyme, secondary metabolites and biomass

**Figure 7:**
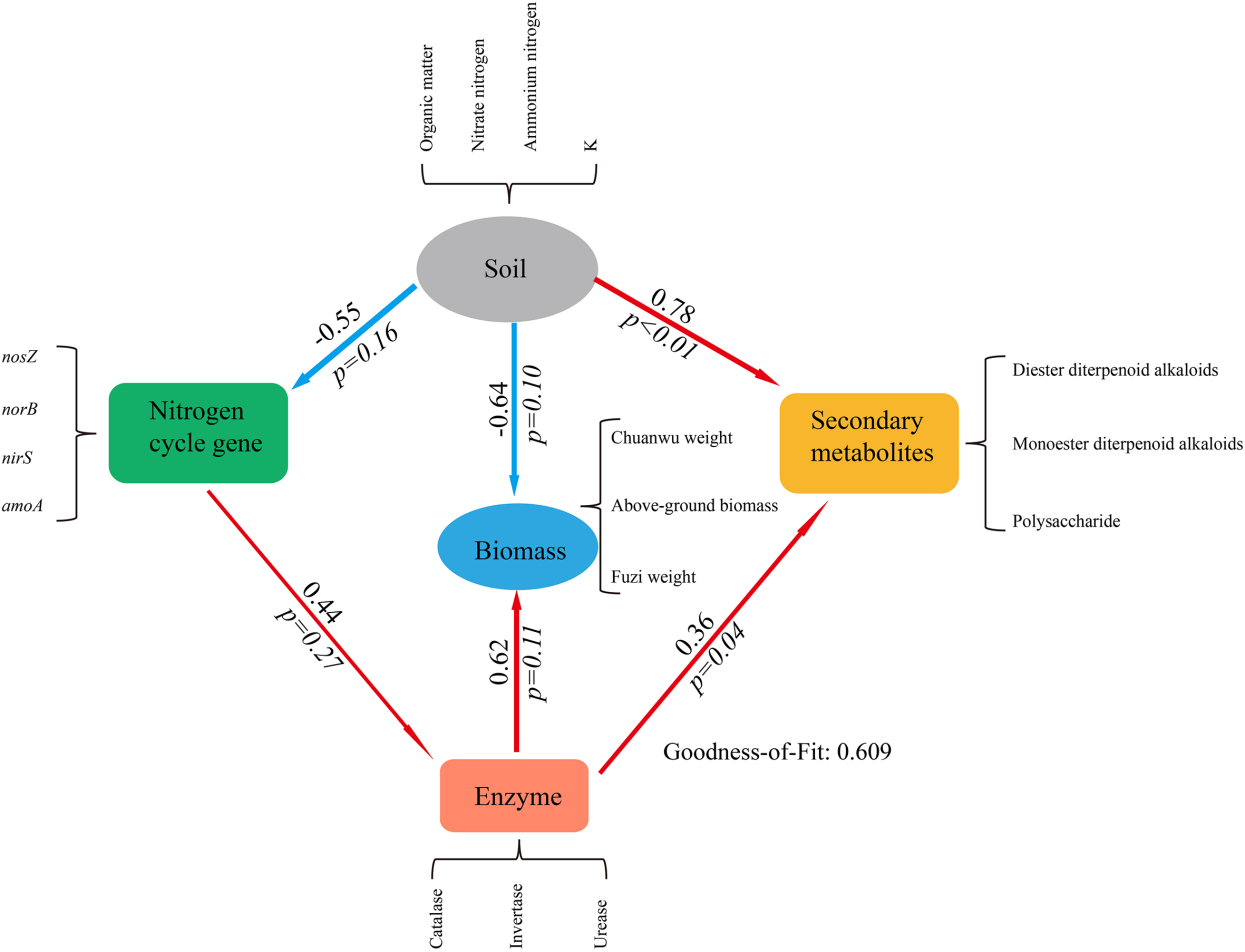
Partial least squares path model (PLS-PM) showing direct and indirect effects of different factors on secondary metabolites and biomass (the number on the arrow describe the magnitude of the path coefficients, and the red and blue colors indicate for positive and negative effects respectively, the *p* value based on t-test. Soil, nitrogen cycle gene, enzyme, secondary metabolites and biomass are latent. The contents in black braces are their explicit. Soil loading > 0.9, nitrogen cycle gene > 0.8, enzyme loading > 0.7, secondary metabolites loading > 0.7, biomass loading > 0.8).

To further study how the herb secondary metabolites and biomass were affected by biotic and abiotic factors during intercropping. Partial Least Squares Path Model (PLS-PM) was used to analyze relationship between soil physicochemical parameters, nitrogen cycle gene, enzyme, and the herb secondary metabolites and biomass (Liao et al., 2019). The results showed that the soil properties were negative effects on the soil nitrogen cycle gene and the plant biomass, but was positive effects on the herb secondary metabolites (*p*< 0.05). The soil enzyme was positive effects on the herb biomass and secondary metabolites (**Figure 7**). The Goodness of Fit test showed that the model is fit. This result showed that the soil probably is the main factor driving changes in the herb secondary metabolites biomass. Unfortunately, due to the limitation of sample size, the model cannot be further optimized. We will make an in-depth study of this issue in the future.

## Discussion

Because of the short growing season (8–10 months) and high profits, the artificial cultivation of *A. carmichaeli* has become rather extensive in China. Under high temperatures and humid conditions, soil-borne diseases, such as southern blight and root rot, commonly occur and strongly decrease the herb yields. To obtain high yields and profits, farmers increased their use of chemical fertilizers and pesticides, which decreased the herb and soil qualities and further threatened sustainable development. Based on the agroclimatic conditions and limited land resources, developing suitable *A. carmichaeli* cropping systems is promising for the potential achievement of high yields and high-quality herbs. The potential function of the five intercropped crops in this study was the improvement in the *A. carmichaeli* microenvironmental conditions. Thus, in this study, we only focused on the intercropping practice effects on the *A. carmichaeli* growth, herb quality, and soil quality.

Farmers are increasingly using intercropping patterns because of their higher productivity, efficient use of resources, and ability to guarantee income (Maitra et al., 2021). For *A. carmichaeli*, 2 years of continuous cropping could result in serious obstacles, which include poor soil fertility, serious plant disease, yield reductions, and a deterioration in quality. In terms of the soil properties, the continuous cropping process remarkably decreases the pH value, changes the nutrient status, and increases the potential pathogen proportions (Xia et al., 2021). In this study, we selected five local crops for intercropping with *A. carmichaeli*. To confirm the potential effects of the different intercropping systems on the herb growth and quality, we conducted this study in two different regions, with continuous cropping and noncontinuous cropping soil. According to the results, intercropping with rice, maize, peanut, sesame, and mung bean can substantially increase the *A. carmichaeli* growth and decrease the disease index, regardless of whether continuous cropping is used or not. These intercropping patterns could alleviate the continuous cropping obstacles of *A. carmichaeli*. Our positive growth improvement results are consistent with previous research on *A. carmichaeli*–rice intercropping systems (Ren et al., 2018). Researchers have also observed the same yield-promoting effect in vegetables–*Ophiopogonis Radix* (Wu et al., 2015), and maize–*Pinellia ternate* (Zhu et al., 2020). However, some researchers have reported that certain intercrops considerably decrease the crop yields, or do not influence them at all (Solanki et al., 2019).

Plants produce additional secondary metabolites to adapt to biotic and abiotic stresses (Jaakola and Hohtola, 2010). In general, these stress conditions are probably beneficial to secondary metabolite accumulations but not to plant growth. Different plant species accumulate special metabolites to adapt to certain environmental factors. In the present study, the different intercrops affected the Fuzi diterpenoid alkaloid and polysaccharide accumulations, and the variation trends for the continuous crop soil were different from those for the noncontinuous crop soil. Researchers have corroborated our results in past studies. The root triptolide content of *Tripterygium wilfordii* was increased under intercropping with Magnolia bark, while it was reduced with *Pinus massoniana* (Ni, 2013). Vegetables–*Ophiopogonis Radix* and maize–*Pinellia ternate* did not substantially affect the qualities of the two medicinal plants (Wu et al., 2015; Zhu et al., 2020). In *Lycium barbarum*–Gramineae systems, the carotenoid, flavonoid, total sugar, and ascorbic acid contents in Wolfberry were increased, while the betaine content was considerably reduced (Zhu et al., 2022).

In our study, the effects of five local intercropping crop patterns on the accumulations of Fuzi polysaccharides, diterpenoid alkaloids were different, which suggests that intercropping could change the medicinal plant quality by influencing the metabolite content; however, the effects are various, and not all the developments are favorable. According to the comprehensive evaluation of the *A. carmichaeli* growth and quality, the best intercropping system was the J-rice, followed by the J-maize, C-peanut, and C-maize. Intercropping with rice, maize, and peanut can obtain higher Fuzi and Chuanwu biomasses, which means higher economic returns. At the *A. carmichaeli* harvest time, only the maize height (about 1.5 m) was higher than the herb height (about 1.2 m), and the heights of the other four crops were lower than that of the herb: rice: about 80 cm; mung bean: about 40 cm; sesame: about 80 cm; peanut: about 25 cm. The effects of the competitive radiation absorption from the four lower crops to the herb could be negligible. Moreover, the intercropping with the five local crops decreased the herb root/shoot ratios and disease indexes, which means that the intercropping practices provided better growth conditions for *A. carmichaeli*. Thus, the intercropping practice improvement in the herb growth and quality may be attributable to the soil quality enhancements.

Most of the intercropping systems could improve the soil quality by assuring the stronger utilization and removal of the available soil nutrients, as well as higher soil biological functions. However, there were variations in the specific physicochemical properties, enzyme activities, and microbial abundances in the rhizosphere soil of the different intercropping patterns and years. All these characteristics influenced each other and provided specific soil microenvironmental conditions for the plant. Researchers primarily use the pH, organic matter, macroelements (nitrogen, phosphate, and potassium), and soil enzymes as the soil quality and fertility indicators. Catalase relieves the toxic effect of hydrogen peroxide, and urease catalyzes and decomposes urea into carbon dioxide, water, and ammonia and supplies available nitrogen nutrients (Song et al., 2021).In previous reports, researchers indicate that conservative monocultures of *A. carmichaeli* may decrease the soil pH, which increases the continuous cropping decline, which, in turn, inhibits the nitrogen and potassium utilization and further affects the soil microorganism communities (Xia et al., 2021). According to the results of this study, the intercropping patterns remarkably increased the soil pH, and the increments in the continuous soil were higher than those in the first-year culture soil.

In the present study, we did not use any fertilizers. From June to July, both *A. carmichaeli* and the five local crops are in vigorous growth stages, and almost none produce aboveground litter. After harvesting, we removed all the *A. carmichaeli* aboveground parts because a high number of alkaloids likely decreases the soil quality. Thus, the only plant residue input in the present study was the root residues of the *A. carmichaeli* and intercropping crops. According to our results, there were substantially higher root biomasses in the five intercropping systems, which means that there was more root residue in the soil and potentially improved soil organic matter. However, the soil organic matter contents in the noncontinuous intercropping patterns were increased, while they were decreased in the continuous intercropping systems. Although the soil NH_4_^+^-N and NO_3_^-^-N had different variant trends in these intercropping systems, the C/N ratios of all of them decreased, which indicates a higher decomposition rate by the soil microbe decomposer community (Cong et al., 2012). The increments of most of the nitrogen-cycling-gene abundances also confirmed this result. The *amoA*, *nxrA*, *nirS*, *norB*, *narG*, *nosZ*, and *nifH* abundances in these intercropping patterns were remarkably increased or not affected, except for the *nxrA* abundance in the J-maize, *norB*, and *nifH* abundances in the J-rice, and *nosZ* abundance in the C-sesame, which decreased. The increments of these gene abundances demonstrated that the greater the ammonia conversion, the higher the nitrification and denitrification rates. Moreover, in comparison with the control groups, most of the four enzyme activities were increased, except for in a few intercropping systems. The increase in these soil enzyme activities contributes to an increase in the overall nutrient cycling in the field.

Moreover, the intercropping with the five local crops did not strongly affect the soil K^+^, Si, Mn^2+^, Zn^2+^, Cu^2+^, and Mo^6+^ levels, and the effects on the phosphates, Ca^2+^, Mg^2+^, and Fe^2+^, as well as other nutrients, were different. In a sugarcane–soybean system, the soil pH was decreased, the organic carbon, total nitrogen, and NH_4_^+^-N contents were increased, and the researchers did not observe any substantial effects in the total K, AP, AK, and NO_3_^-^-N (Malviya et al., 2021). The total nitrogen, available phosphorus, and organic matter concentrations and urease activity in the maize soil of a maize–soybean intercrop were substantially increased; however, the soil pH did not change (Fu et al., 2019). Peach–Morchella intercropping substantially increased the organic matter, available potassium, and available zinc contents, as well as the catalase, sucrase, cellulase, and urease activities, except for the pH value (Song et al., 2021). These results indicated the effects of different intercropping systems on the specific soil properties were various. According to the comprehensive evaluation of the *A. carmichaeli* soil quality in the present study, the best intercropping system was the J-maize, followed by the J-rice, C-sesame, and C-peanut. The differences in the soil properties between Jiangyou and Chenggu are attributable to the lower original soil quality from the continuous cropping soil in Chenggu, and especially to the different soil microbial populations (Xia et al., 2021).

Soil microbial communities play a significant role in soil physiochemical and biological functions by secreting extracellular enzymes and releasing intracellular enzymes into the soil. In turn, plants modify the microbial abundance and diversity through their root exudations and interactions (Blaise et al., 2021). In this study, the 16S rDNA and ITS rDNA abundances detected by RT-PCR in the six intercropping systems were increased, except for the ITS abundance of the J-maize, which means that the total fungus and bacteria in these intercropping systems were increased. Both maize and rice intercropping could not substantially alter the soil microbial α diversity; however, it strongly influenced the microbial community structure. In the two intercropping systems, the potential functions of 28 potential beneficial genera included the following: the generation of IAA (Bal et al., 2013), nitrogen fixation (Hayat et al., 2010; Shahrajabian et al., 2021), soil mercury content reduction (Ustiatik et al., 2022), the degradation of polycyclic aromatic hydrocarbons (Zhao et al., 2021), antibacterial functions (Mosquera et al., 2020), antimycotic functions (De Brito et al., 2022), aluminum tolerance (Silambarasan et al., 2022), salt and alkali resistance (Wang et al., 2022), phosphate solubilization (Chhetri et al., 2021; Saadouli et al., 2021), siderophores (Jamalzadeh et al., 2021; Nie et al., 2022), nitrification (Peng et al., 2021), etc. In the control group, the potential functions of the 15 potentially beneficial genera were as follows: the nitrogen cycle (Zhang et al., 2022), antibiotic functions (Nakkeeran et al., 2021), heavy metal resistance (Zhai et al., 2020; Sharma et al., 2022), phosphate solubilization (Huang et al., 2021; Duan et al., 2022), improved photosynthesis (Katsenios et al., 2021), nitrification (Li et al., 2022), etc. Six of the harmful genera were pathogens (Liu et al., 2020b; Na et al., 2021; Tancos et al., 2021; Kaari et al., 2022; Patane et al., 2022). These beneficial genus microorganisms could improve the intercropping soil qualities.

## Conclusions

In conclusion, the intercropping with the five local crops substantially improved the *A. carmichaeli* agronomic characteristics, such as the stem diameter, aboveground biomass, Fuzi biomass, Chuanwu biomass, and total biomass, and decreased the disease occurrence. The intercropping also increased the herb polysaccharide content and altered the diterpenoid alkaloids. According to the comprehensive evaluation of the *A. carmichaeli* growth and quality, as well as the soil quality, under these treatments, the best intercropping systems were the *A. carmichaeli* intercropping with rice, maize, and peanut. The soil quality improvement was mainly attributable to the preferable soil function and greater abundance of beneficial bacterium genera.

## Data availability statement

The datasets presented in this study can be found in online repositories. The names of the repository/repositories and accession number(s) can be found below: https://www.ncbi.nlm.nih.gov/bioproject/PRJNA907203.

## Author contributions

LC, CL, and PY contributed to the conception, investigation, conceptualization and design of this study. CL and PY performed the experiments, writing original draft and analyzed the data. LC, ZL, and JZ performed the statistical analysis and revised the paper. LC and GZ: supervision, project administration, and funding acquisition. All authors have read and approved the final manuscript for publication.

## Supplementary material

Supplementary Figure 1 Strip-intercropping experimental location and planting diagram. **(A)**: location of the experimental sites in China; **(B)**: location of the experimental site in Jiangyou and Chenggu; **(C)**: strip-intercropping mode, take maize as an example; **(D)**: monocropping mode.

Supplementary Figure 2 Venn diagram of species under three treatments. Note: **(A)**: the phylum level; **(B)**: the genus level; **(C)**: the species level.

Supplementary Figure 3 Relative abundances of soil microorganisms at the taxonomic level. Note: The y-axis is truncated between 0-4%, and 4%-100% of the y-axis is all bacteria.

Supplementary Figure 4 Relative abundances of the top 15 microorganisms in each soil sample at the phylum level. Note: **(A)** is the general overview, and **(B)** is the truncation of y-axis of figure at between 90%-100%.

Supplementary Figure 5 Relative abundances of the top 15 microorganisms in each soil sample at the genus level. Note: y-axis is truncated between 0-60%, and 60%-100% is Others.
