# Supplementary figures and images for: Strip intercropping with local crops increased Aconitum carmichaeli yield and soil quality
Source: Front Plant Sci. 2023 Mar 3;14:1147671. doi: 10.3389/fpls.2023.1147671 (PMC10020659; doi:10.3389/fpls.2023.1147671)

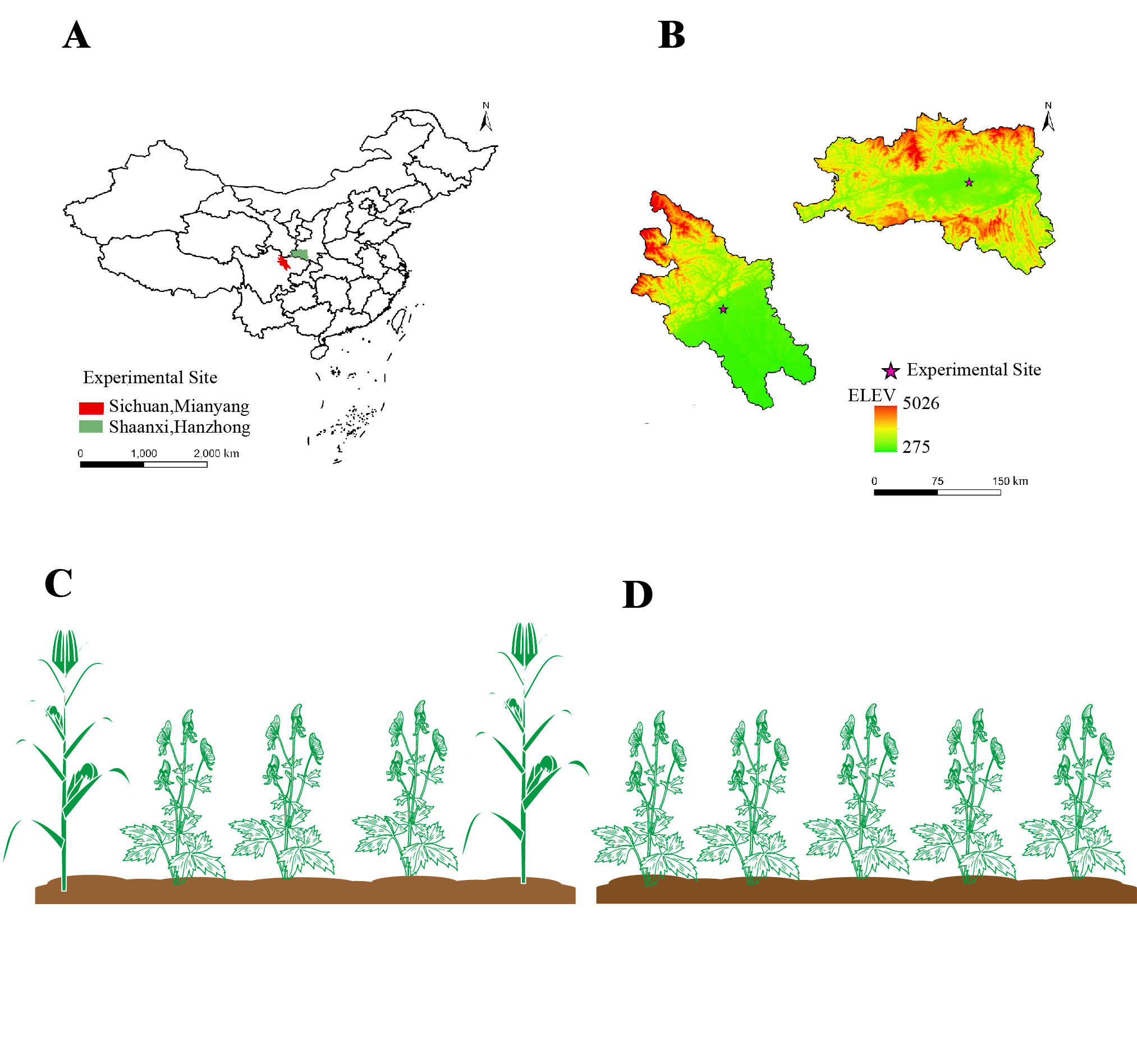

Supplement: Supplementary Figure 1 — Strip-intercropping experimental location and planting diagram. (A): location of the experimental sites in China; (B): location of the experimental site in Jiangyou and Chenggu; (C): strip-intercropping mode, take maize as an example; (D): monocropping mode. [file Image_1.jpeg]

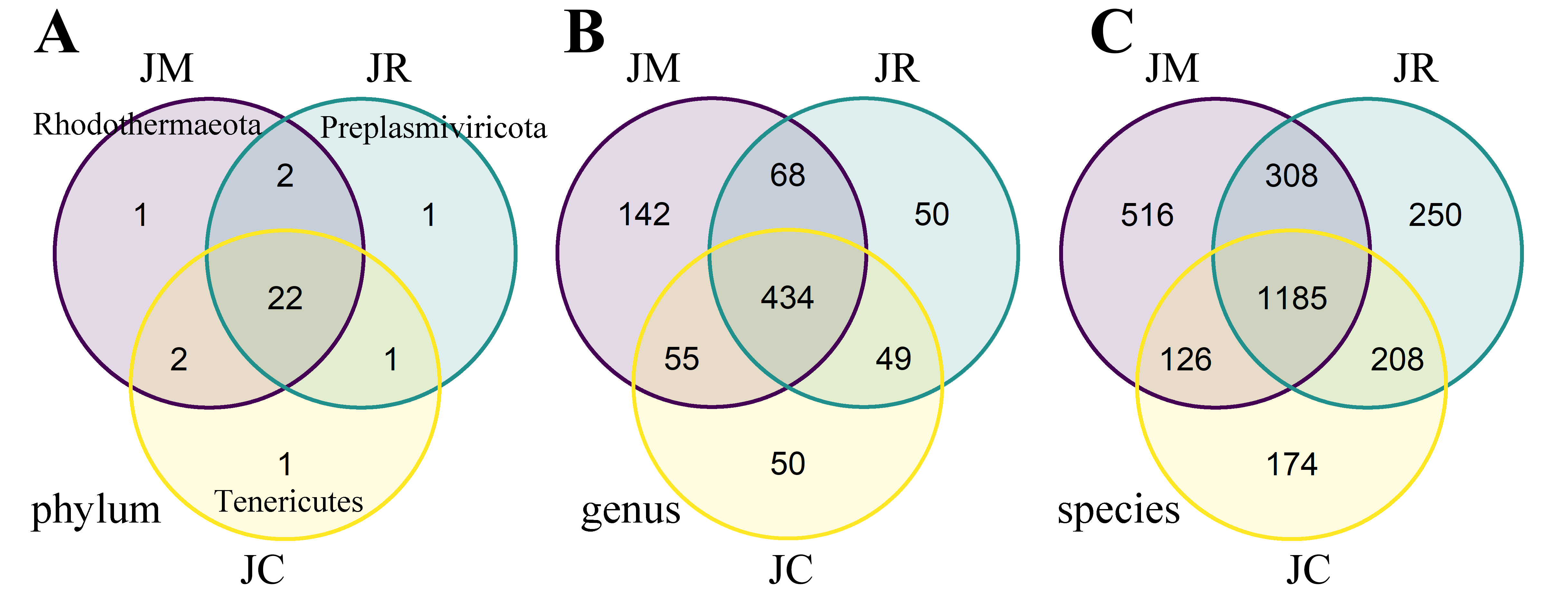

Supplement: Supplementary Figure 2 — Venn diagram of species under three treatments. Note: (A): the phylum level; (B): the genus level; (C): the species level. [file Image_2.jpeg]

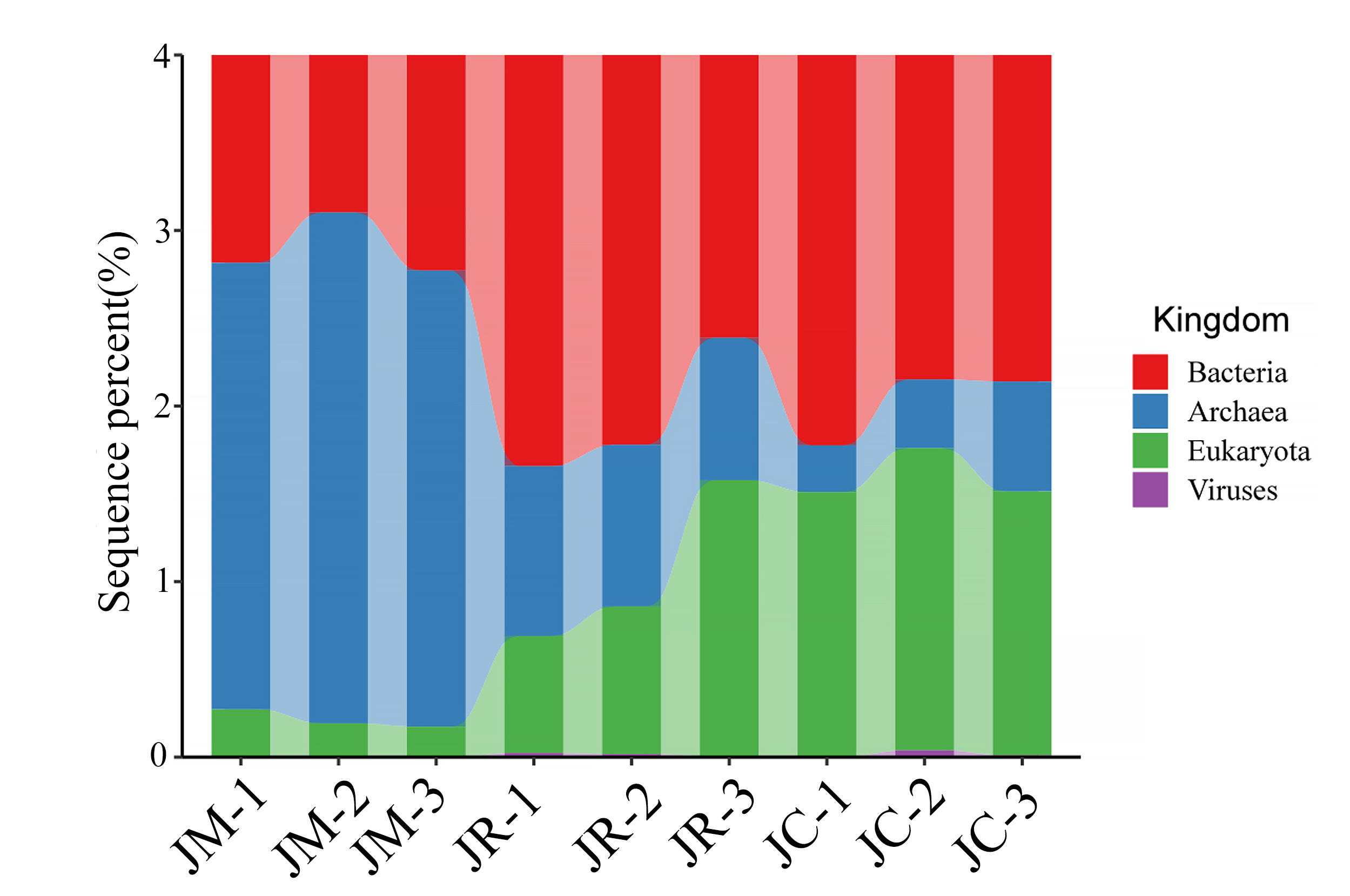

Supplement: Supplementary Figure 3 — Relative abundances of soil microorganisms at the taxonomic level. Note: The y-axis is truncated between 0-4%, and 4%-100% of the y-axis is all bacteria. [file Image_3.jpeg]

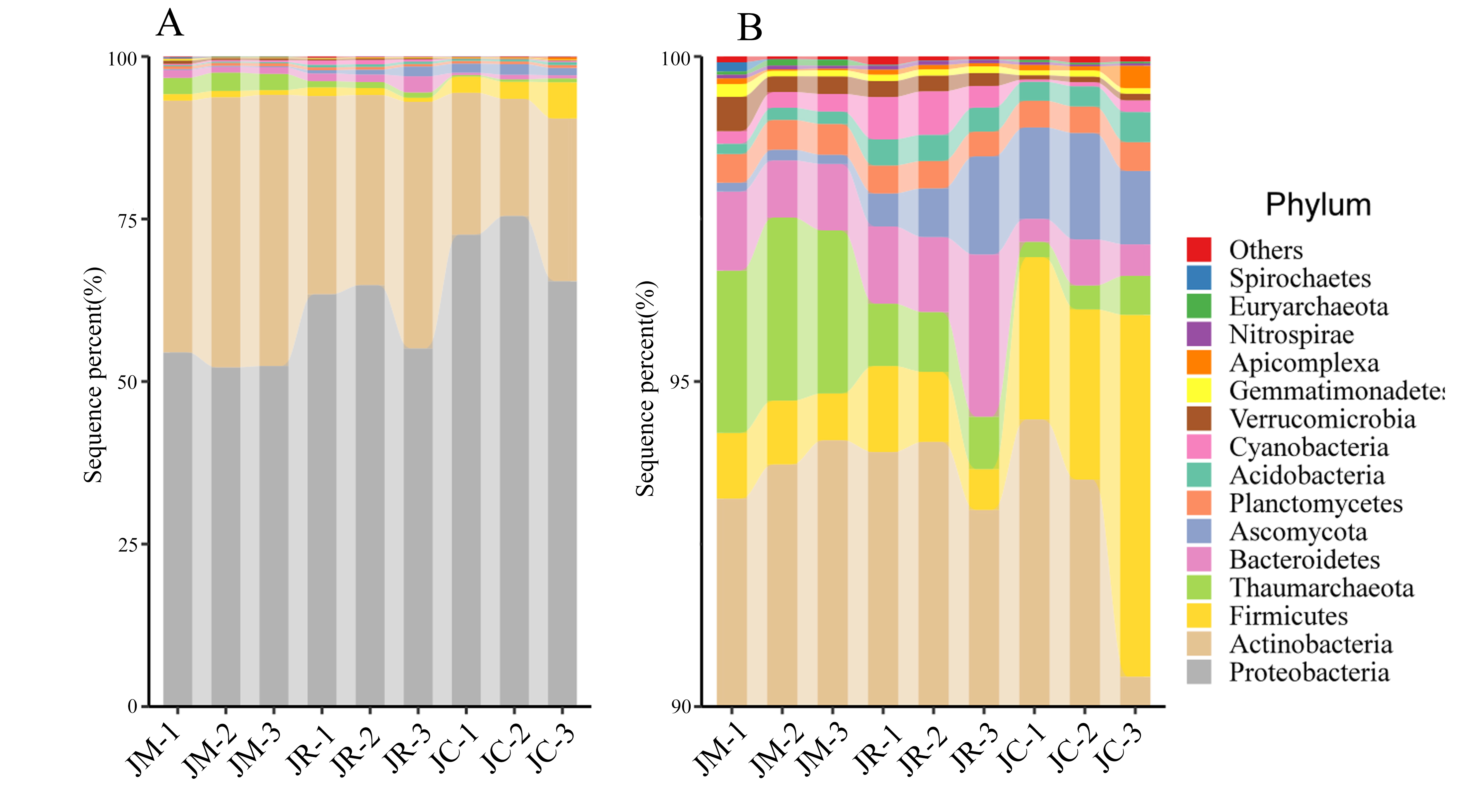

Supplement: Supplementary Figure 4 — Relative abundances of the top 15 microorganisms in each soil sample at the phylum level. Note: (A) is the general overview, and (B) is the truncation of y-axis of figure at between 90%-100%. [file Image_4.jpeg]

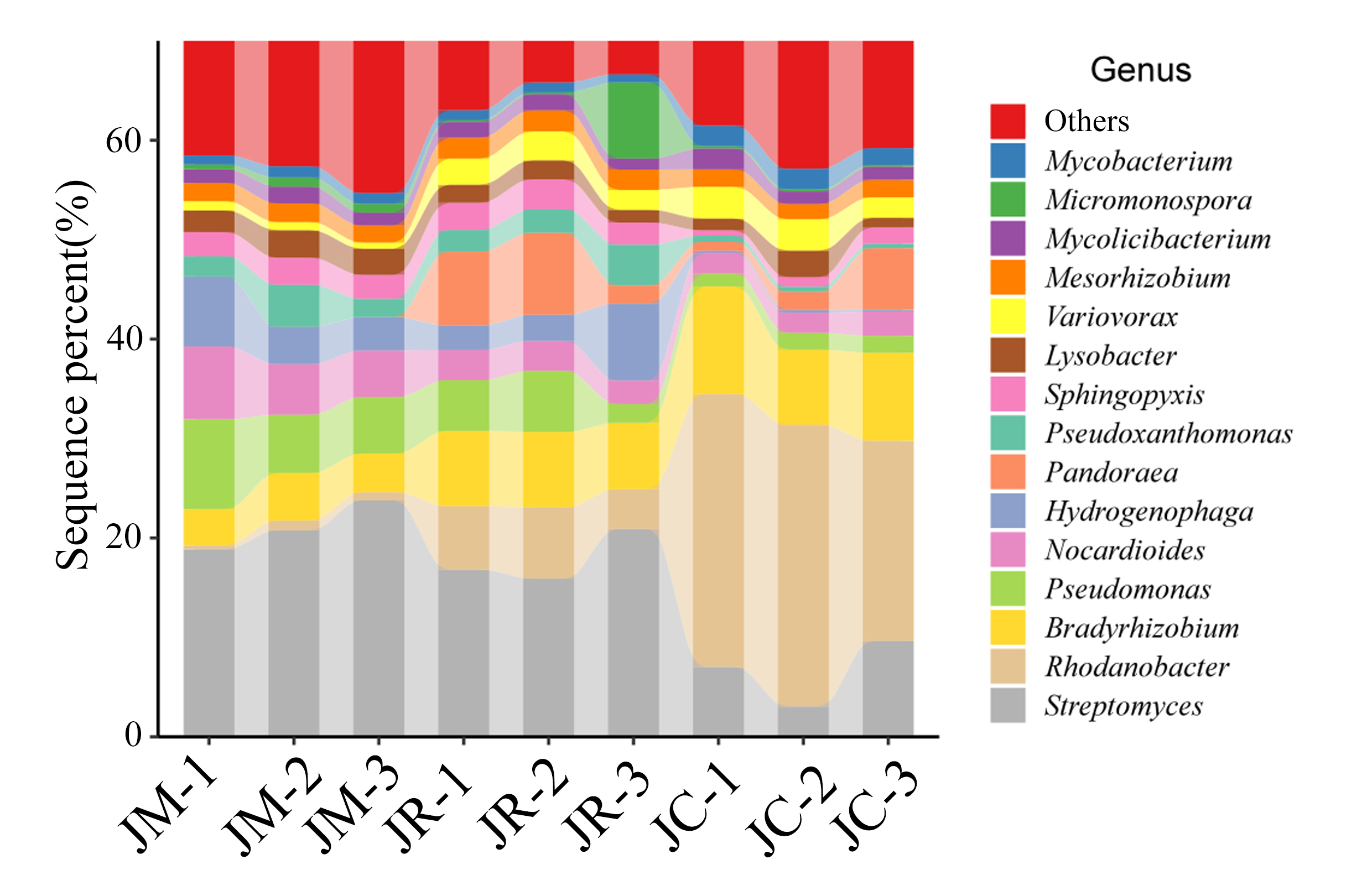

Supplement: Supplementary Figure 5 — Relative abundances of the top 15 microorganisms in each soil sample at the genus level. Note: y-axis is truncated between 0-60%, and 60%-100% is Others. [file Image_5.jpeg]
